# Unmasked renal impairment and prolonged hyperkalemia after unilateral adrenalectomy for primary aldosteronism coexisting with primary hyperparathyroidism: report of a case

**DOI:** 10.1007/s00595-013-0813-0

**Published:** 2013-12-17

**Authors:** Yatsuka Hibi, Nobuki Hayakawa, Midori Hasegawa, Kimio Ogawa, Yoshimi Shimizu, Masahiro Shibata, Chikara Kagawa, Yutaka Mizuno, Yukio Yuzawa, Mitsuyasu Itoh, Katsumi Iwase

**Affiliations:** 1 Department of Endocrine Surgery, Fujita Health University School of Medicine, 1-98 Dengakugakubo, Kutsukake-cho, Toyoake, Aichi 470-1192 Japan; 2 Clinical Pharmacotherapeutics I, Faculty of Pharmacy, Meijo University, Nagoya, Japan; 3 Department of Nephrology, Fujita Health University School of Medicine, Aichi, Japan; 4 Division of Endocrinology and Metabolism, Department of Internal Medicine, Fujita Health University School of Medicine, Aichi, Japan

**Keywords:** Primary hyperaldosteronism, Hyperparathyroidism, Kidney impairment

## Abstract

We herein report the case of a patient with critical hyperkalemia after unilateral adrenalectomy (ADX) for aldosterone-producing adenomas, which were coexisting with primary hyperparathyroidism. A right adrenal tumor oversecreting mineral corticoid was identified in a 62-year-old female whose kidney function had been impaired due to primary hyperaldosteronism and hyperparathyroidism. The ADX improved her hypertension with normalization of the plasma aldosterone concentration, but without adequately increasing her plasma renin activity. Her eGFR further decreased postoperatively, hyperkalemia appeared and the serum potassium level rose to 6.3 mEq/L at 3 months after ADX. Then, treatment with calcium polystyrene sulfonate jelly was started. Eight months after ADX, a left lower parathyroidectomy was performed, and the serum calcium and intact parathyroid hormone levels decreased to the normal range. The hyperkalemia was difficult to control within 20 months postoperatively without treatment with calcium polystyrene sulfonate jelly or hydrocortisone. This suggests that unmasking the renal impairment and relative hypoaldosteronism after ADX might induce critical hyperkalemia.

## Introduction

Primary aldosteronism (PA), characterized by autonomous hypersecretion of aldosterone, results in secondary hypertension as a result of excessive sodium retention and potassium excretion. Furthermore, recent studies have reported that PA may lead to hyperfiltration in the glomeruli of the kidneys [1, 2], and may therefore induce direct renal structural damage independent of the hypertension [3–7]. Significant histological damage of the kidneys in patients with PA has also been described [8].

Primary hyperparathyroidism (PHPT) is a relatively common endocrine disorder. About 90 % of cases are caused by a single gland adenoma, and fewer than 10 % are caused by multiglandular disease. Oversecretion of parathyroid hormone (PTH) causes hypercalcemia, resulting from inappropriate bone absorption and the reabsorption of calcium from the distal tubules in the kidneys. It is considered that excess PTH impairs renal function and reduces the glomerular filtrating rate [9, 10].

In this report, we present the case of a patient who was revealed to have critical hyperkalemia which might have been caused in the setting of unmasked renal impairment and relative hypoaldosteronism after ADX for an aldosterone-producing adenoma (APA), coexisting with PHPT.

## Case report

A 62-year-old female was examined for hypertension that had been refractory to medication for 5 years, in a local hospital. The laboratory data suggested hyperaldosteronism, and an abdominal CT scan identified a right adrenal tumor. Since she also had a history of urinary calculi attacks, the intact PTH level and serum Ca level were examined; both of which were found to be beyond the normal range. She was referred to our hospital for further evaluation and treatment.

**Fig. 1:**
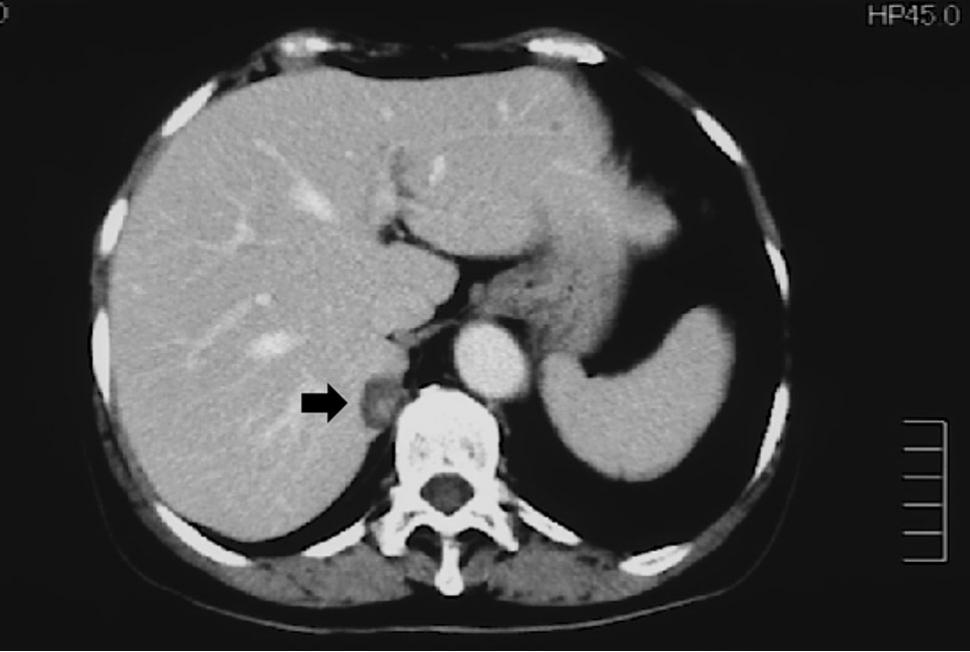
Computed tomography of the abdomen showed a right adrenal tumor of 2.0 cm in diameter (*arrow*)

**Fig. 2:**
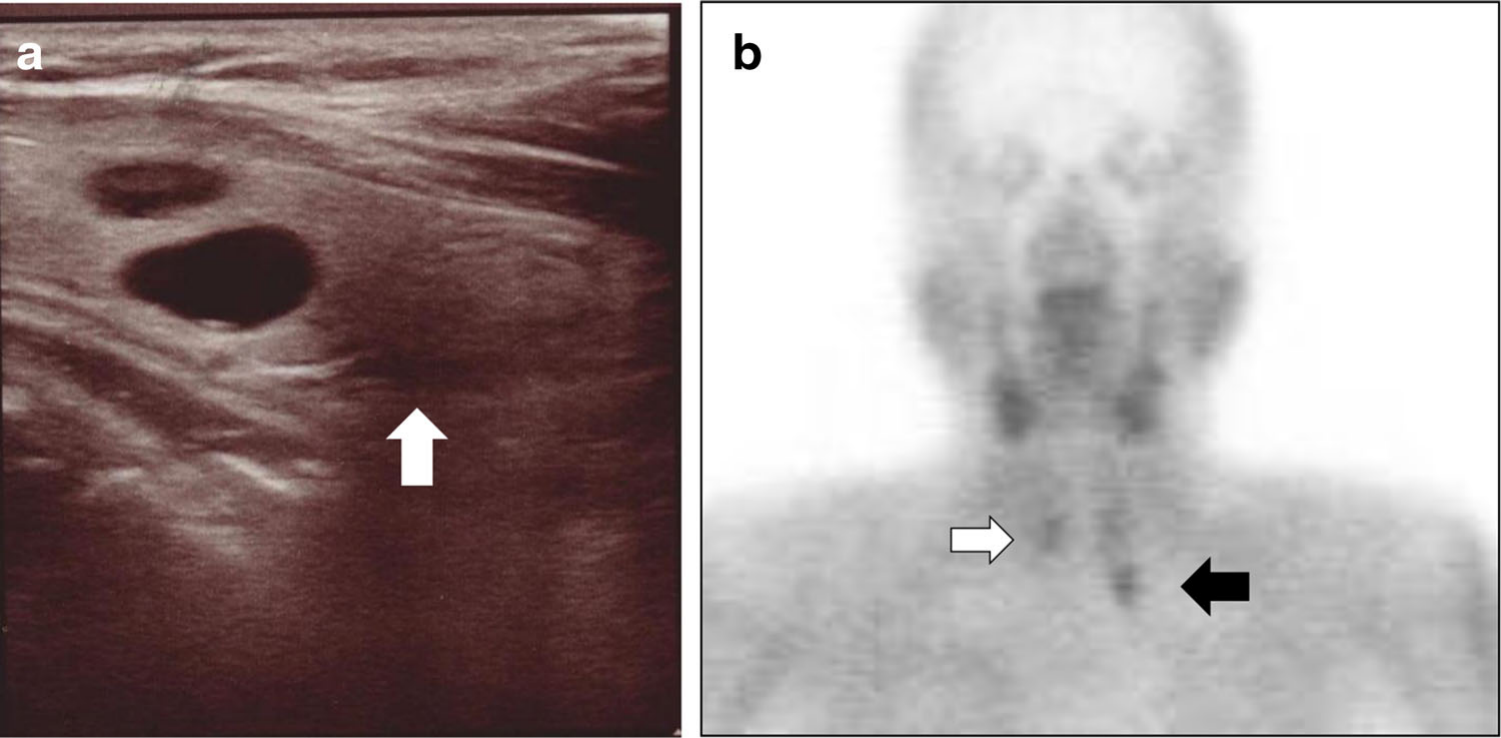
**a** Neck US exhibited a low echoic mass behind the lower pole of the patient’s left thyroid lobe (*white arrow*). **b** The delayed phase of the ^99m^Tc-MIBI scintigram indicated abnormal uptake in the left *(arrow*) and right (*open arrow*) neck regions (**a**)

The laboratory data concerning the adrenal and parathyroid function are shown in Table 1. At admission, the patient’s blood pressure was 168/107 mmHg. Before admission, the local hospital had given her the antihypertension drugs, nifedipine and bunazosin, but had not administered a mineral corticoid receptor antagonist or a potassium preparation. The aldosterone/renin ratio (ARR) was 570 according to the Captopril suppression test, and the plasma renin activity (PRA) remained suppressed (1.0 ng/mL/h) in the furosemide-upright test, indicating the autonomous secretion of aldosterone. An abdominal CT scan showed a right adrenal tumor measuring 2 cm in diameter (Fig. 1). In the ACTH-loading adrenal venous sampling test (AVS), the level of plasma aldosterone concentration (PAC) was 220,000 pg/mL, and the lateralized ratio calculated by the aldosterone/cortisol ratio (A/C) of the right adrenal vein to the A/C ratio of the left adrenal vein was 15.2. Therefore, the patient was diagnosed with APA in the right adrenal, because these results met the criteria for APA from The Japan Endocrine Society [11]. Hyperparathyroidism was also diagnosed upon reference to our hospital. The intact PTH level was 785.7 pg/mL, and the serum calcium and phosphate levels were 10.7 and 3.5 mg/dL, respectively. The neck US showed a low echoic mass behind the lower pole of her thyroid left lobe (Fig. 2a). The delayed phase of a ^99m^Tc-MIBI scintigram showed abnormal uptake in the left and right sides of her neck (Fig. 2b). She did not have any other lesions or any familial history related to MEN1.

**Table 1:** Results of blood tests for adrenal and parathyroid function

		Normal range
Adrenaline	29 pg/mL	<100
Noradrenaline	367 pg/mL	100–450
Dopamine	<5 pg/mL	<5
ACTH	38.0 pg/mL	7.2–63.3
Cortisol	14.4 mg/dL	4.0–19.3
Plasma renin activity	<0.1 ng/mL/h	0.3–2.9
Plasma aldosterone	901 pg/mL	35.7–240.0
Intact PTH	787.5 pg/mL	15.0–68.6
Creatinine	1.18 mg/dL	0.4–0.7
BUN	24.1 mg/dL	8.0–22.0
eGFR	36.6 mL/min/1.73 m^2^	>60
Na	145 mEq/L	138–146
K	3.1 mEq/L	3.6–4.9
Cl	109 mEq/L	99–109
Ca	10.9 mg/dL	8.7–10.3
P	2.4 mg/dL	2.5–4.7
Osteocalcin	24 pg/mL	2.5–13.0
BAP	31.1 μg/L	3.8–22.6
NTx	286.5 nmol BCE/mmol CRE	14.3–89.0

**Fig. 3:**
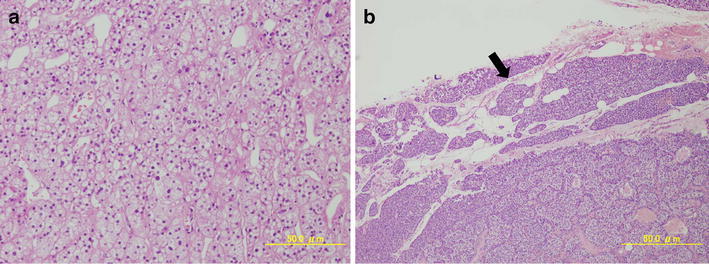
**a** Microscopy of the right adrenal tumor showed a cortical adenoma (×100): hematoxylin–eosin staining. **b** Microscopy of the left parathyroid tumor showed a cortical adenoma containing a normal rim (*arrow*): hematoxylin–eosin staining

We initially performed laparoscopic right ADX for the APA. The diagnosis of adrenal cortical adenoma was confirmed by a histological examination (Fig. 3a). Her hypertension improved gradually, and the antihypertensive drugs were discontinued 3 months after ADX.

**Fig. 4:**
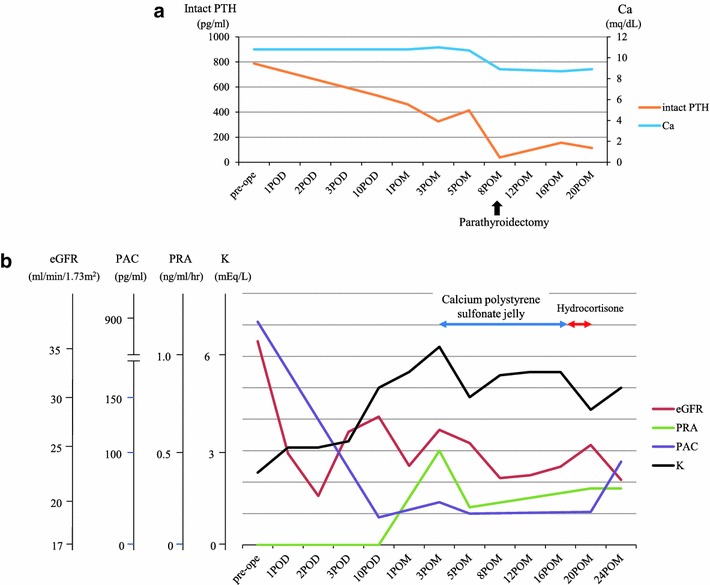
**a** The intact PTH level decreased from 787.5 to 413.1 pg/mL after ADX, but the serum Ca level did not decrease (10.8–11.0 mg/dL). Parathyroidectomy was performed 8 months postoperatively. The intact PTH and serum Ca levels were normalized (37.9 pg/mL and 8.9 mg/dL each) after parathyroidectomy. **b** The eGFR was 46.6 mL/min/1.73 m^2^ preoperatively (eGFR classification stage 3b) and declined to below 30 mL/min/1.73 m^2^ (stage 4) after ADX. The potassium level increased and reached 6.3 mEq/L at 3 months postoperatively. The administration of calcium polystyrene sulfonate jelly and hydrocortisone sequentially decreased the potassium level to 4.3 mEq/L at 20 months postoperatively. The PRA stayed relatively low (0.1–0.5 ng/mL/h each) and the PAC also dropped to a relatively low level (44.4–49.9 pg/mL) by 24 months postoperatively. *POD* postoperative day after ADX, *POM* postoperative month after ADX

Since the PTH level gradually decreased after ADX, but the serum Ca level stayed high (Fig. 4a), surgical treatment was performed for PHPT 8 months after ADX. The left lower parathyroid gland was resected, and the lesion in the right lower pole was identified to be a thyroid nodule by bilateral neck exploration, which might have been responsible for the uptake of ^99m^Tc-MIBI. The other three parathyroid glands were confirmed to be normal macroscopically during the operation. From the histopathological examination, the resected left lower parathyroid gland, measuring 17 × 12 × 5 mm and weighing 773 mg was diagnosed to be adenoma (Fig. 3b). The postoperative intact PTH level and serum Ca level significantly decreased to be within the normal range (37.9 pg/mL and 8.9 mg/dL, respectively).

## Changes in the eGFR, PRA, PAC and serum potassium levels after ADX

The changes in the eGFR, PRA, PAC and serum potassium levels are shown in Fig. 4b. The eGFR before ADX was 36.6 mL/min/1.73 m^2^, which is stage 3b in the eGFR classification of the KDIGO 2012 Clinical Practice Guideline, suggesting that her renal function was impaired. The eGFR further decreased to 20.2 mL/min/1.73 m^2^ (stage 4) 2 days after the ADX. This renal impairment did not improve even after parathyroidectomy. The postoperative PRA remained at a low level (0.1–0.5 ng/mL/h) and the PAC declined to a relatively low level (44.4–49.9 pg/mL) for a while, although it stayed above the lower limits of the normal range. Likewise, the serum potassium level increased after postoperative day 10 and was 6.3 mEq/mL at its maximum 3 months after ADX in the outpatient department. Treatment with calcium polystyrene sulfonate jelly was started. Ultimately, 20 mg/day of hydrocortisone was given instead of calcium polystyrene sulfonate jelly after taking into account the low zona glomerulosa function via resulting from the suppressed plasma renin levels after surgery. The hydrocortisone was decreased and finally discontinued because the potassium level fell to 4.7 mEq/L, indicating the resolution of the critical hyperkalemia, and the PAC level increased.

## Discussion

We herein presented the case of a patient with renal impairment whose Cr level increased after surgical treatment for APA. A recent study documented a higher prevalence of renal impairment in patients with PA as compared to those with primary hypertension [12], suggesting that there is direct renal damage by excess aldosterone. Experimental studies have demonstrated that aldosterone may produce direct and rapid vasoconstrictor actions in the efferent renal arteriole [13], or may mediate vascular remodeling in part via increased oxidative stress [14, 15]. Moreover, several clinical studies reported a decline of kidney function in the subset of patients with APA after medical or surgical treatment [1, 16]. This suggests that normalization of the excess secreted aldosterone may contribute to improving the intraglomerular pressure, and thereby serve to unmask actual kidney impairment [16].

In our patient, the elevation of the serum Cr level and decline of the eGFR started to be observed on postoperative days 1–2, although the patient had originally showed stage 3b renal impairment in the eGFR classification before ADX. Although these conditions may have arisen partly as a direct effect of anesthetic or surgical stress, as suggested by previous reports, it can be considered more likely that actual renal impairment was unmasked after relief of the hyperfiltration in the glomeruli by the surgical treatment of the APA. A decrease in the eGFR is most likely to take place after around 6 months of treatment for primary aldosteronism [5], but in our case, this decline developed approximately 8 months after ADX, but persisted thereafter. It is considered that this kind of renal impairment would result from the influence of not only hypercalcemia due to PHPT, but also due to APA.

Likewise, it has been reported that the renal function is sometimes transiently impaired just after parathyroidectomy in patients with hyperparathyroidism [9, 17]. In our case, it was also observed after parathyroidectomy (Fig. 4a). In an animal model, PTH was also demonstrated to increase the renal blood flow by exerting vasodilatory effects in pre-glomerular vessels and vasoconstrictor actions in the efferent renal arteriole [9]. Moreover, Evenepoel et al. [17] speculated that reversal of this effect, followed by parathyroidectomy, may result in an acute deterioration of renal function.

Although hyperkalemia is one of the possible complications after ADX for APA that should be kept in mind, it is commonly mild and transient [18]. However, there have been three case reports of documented postoperative prolonged hyperkalemia treated with fludrocortisone [19–21]. Two of these reports indicated that the prolonged hyperkalemia was due to hypoaldosteronism caused by the zona glomerulosa function occurring as a result of suppressed plasma renin levels after surgery [19, 20]. In our case, the postoperative PRA was low (0.1–0.5), but the PAC (44.4–49.9) stayed within the normal range. This might have reflected the state of relative ‘hypoaldosteronism’ that induced the hyperkalemia. Fisher et al. [22] analyzed the incidence of hyperkalemia after adrenalectomy in a large series (*n* = 110) of APA cases. They reported that six patients (5 % of the total cohort) presented with prolonged postoperative hyperkalemia (serum potassium level >5 mEq/L), and there were three patients (2.5 %) who manifested hyperkalemia of more than 6 mEq/L, in the same manner as that observed in our case. However, as they mentioned in that study, the proportion of patients who were hypokalemic before surgery was very high (92 %), implying that there were more severe APA cases, and the incidence might have been overestimated by a possibly greater and more prolonged degree of suppression of aldosterone production in the contralateral adrenal gland. However, the study also mentioned that, in their multivariate analysis, the preoperatively decreased eGFR and serum Cr levels, as well as the increased postoperative Cr and microalbuminuria, remained significant predictors of hyperkalemia, indicating that impaired renal function appears to be a strong predictor of hyperkalemia. This finding supports the notion that renal impairment itself due to APA also might contribute to postoperative prolonged hyperkalemia. Thus, the development of critical hyperkalemia, as in our case, would be caused in the setting of unmasked renal impairment and relative hypoaldosteronism.

We started to administer calcium polystyrene sulfonate jelly to improve the critical hyperkalemia before the treatment of the PHPT. Although intake of calcium polystyrene sulfonate may increase the serum calcium level [23], the resulting levels are not likely to have been influenced by the drug in our case (Fig. 4a, b). However, in general, one must be very careful about changing the serum calcium level when administering this drug to patients with hypercalcemia.

Our patient’s hyperparathyroidism was considered to be PHPT, because the affected gland was diagnosed to be a single adenoma based on the intraoperative and histopathological findings. Several studies have reported PHPT in patients with primary aldosteronism [24–28]. In patients with MEN1 mutations, the most frequently encountered features are PHPT, pancreatic endocrine tumors and pituitary adenomas, but adrenal cortical adenoma is also found at a frequency of around 9–45 % [29]. Most of these adrenal cortical adenomas have been considered non-functional, but recent studies have suggested that APA could be a more common component of the MEN1 adrenal phenotype than previously considered [30]. However, the coexistence of PHPT and PA in our case would be unrelated to MEN1, because parathyroid glands affected by MEN1 are usually multiple and do not contain a normal rim in the histopathological findings.

Aside from disorders in the MEN1 gene, PA and hyperparathyroidism may concomitantly interact with each other. The expression of the type 1 PTH receptor in the aldosterone-producing adrenocortical nodules and of the mineralocorticoid receptor in the nuclei of parathyroid cells has been described [27]. PTH was reported to stimulate the secretion of aldosterone in vitro in a concentration-dependent manner [31]. Moreover, it was demonstrated that, in the setting of experimental aldosteronism, the urinary and fecal calcium excretion was increased with the increase in the PTH level, and these were attenuated or abrogated by treatment with a mineralocorticoid receptor antagonist [32, 33]. A recent study presented clinical data indicating that PA contributed to secondary hyperparathyroidism, and that secondary hyperparathyroidism could be improved by PA treatment [34]. In our case, we interpreted the cause to be the single adenoma of the parathyroid gland in PHPT based on the histopathological findings. However, it cannot be completely ruled out that the direct damage to the kidneys by PA partly induced secondary (renal) hyperparathyroidism, or that aldosterone and PTH interacted with each other, because the PTH level was decreased after ADX.

In conclusion, the present report suggests that unmasking renal impairment after ADX and relative hypoaldosteronism might induce critical hyperkalemia. Careful observation is required after ADX, especially in patients with other complications which may cause renal impairment, such as PHPT.
